# Patterns of Human Plague in Uganda, 2008–2016

**DOI:** 10.3201/eid2309.170789

**Published:** 2017-09

**Authors:** Joseph D. Forrester, Titus Apangu, Kevin Griffith, Sarah Acayo, Brook Yockey, John Kaggwa, Kiersten J. Kugeler, Martin Schriefer, Christopher Sexton, C. Ben Beard, Gordian Candini, Janet Abaru, Bosco Candia, Jimmy Felix Okoth, Harriet Apio, Lawrence Nolex, Geoffrey Ezama, Robert Okello, Linda Atiku, Joseph Mpanga, Paul S. Mead

**Affiliations:** Centers for Disease Control and Prevention, Fort Collins, Colorado, USA (J.D. Forrester, K. Griffith, B. Yockey, K.J. Kugeler, M. Schriefer, C. Sexton, C.B. Beard, P.S. Mead);; Uganda Virus Research Institute, Arua, Uganda (T. Apangu, S. Acayo, J. Kaggwa, G. Candini, J. Abaru, B. Candia, J.F. Okoth, H. Apio, L. Nolex, G. Ezama, R. Okello, L. Atiku, J. Mpanga)

**Keywords:** plague, *Yersinia pestis*, bubonic plague, pneumonic plague, Africa, Uganda, West Nile, zoonoses, bacteria, vector-borne infections, United States

## Abstract

Plague is a highly virulent fleaborne zoonosis that occurs throughout many parts of the world; most suspected human cases are reported from resource-poor settings in sub-Saharan Africa. During 2008–2016, a combination of active surveillance and laboratory testing in the plague-endemic West Nile region of Uganda yielded 255 suspected human plague cases; approximately one third were laboratory confirmed by bacterial culture or serology. Although the mortality rate was 7% among suspected cases, it was 26% among persons with laboratory-confirmed plague. Reports of an unusual number of dead rats in a patient’s village around the time of illness onset was significantly associated with laboratory confirmation of plague. This descriptive summary of human plague in Uganda highlights the episodic nature of the disease, as well as the potential that, even in endemic areas, illnesses of other etiologies might be being mistaken for plague.

Plague is a virulent zoonosis caused by the gram-negative bacillus *Yersinia pestis* (*1**,**2*). The organism cycles naturally among rodents and their fleas in areas with conducive ecology across the Americas, Asia, and Africa (*3*). Most human plague cases occur after the bite of an infected flea and manifest clinically as bubonic plague, with rapid onset of fever and painful regional lymphadenopathy (*4*). Infection with the bacteria can sometimes result in a generalized septic illness lacking obvious lymphadenopathy. Pneumonic plague occurs after dissemination of the bacteria from other parts of the body to the lungs or through direct inhalation of infectious droplets into the lungs. Unlike bubonic plague, pneumonic plague can be transmitted from person to person. Outbreaks of pneumonic plague with high human mortality rates can occur in resource-poor settings (*5**–**7*). The mortality rate for untreated infections ranges from ≈65% for bubonic plague to ≈100% for pneumonic plague (*8**,**9*). Early treatment with effective antimicrobial drugs greatly reduces the risk for death (*4**,**8*).

Currently, sub-Saharan Africa accounts for >95% of reported human plague cases worldwide (*10*). The West Nile region in northwestern Uganda encompasses the current plague focus of that country. This densely populated, remote area near the borders of the Democratic Republic of the Congo and South Sudan predominantly has a subsistence agriculture economy. Much of the region lies at 1,000–2,000 m above sea level and experiences 2 main periods of rainfall, with the heaviest precipitation occurring from late August through November (*11*). Living conditions that include close contact with rodents, a burdened healthcare infrastructure, and unreliable stocks of antimicrobial drugs, combined with infected persons delaying seeking healthcare, all contribute to plague illness and death. An understanding of the demographics, geographic distribution, and outcomes of infection is needed to guide prevention programs. Thus, we summarize the epidemiology of human plague in the West Nile region of Uganda during 2008–2016.

## Methods

Data on human plague cases were collected as part of an ongoing collaboration between the Uganda Virus Research Institute (UVRI), the Ugandan Ministry of Health, and the US Centers for Disease Control and Prevention (CDC) to enhance education, clinical detection, laboratory diagnostic capacity, treatment, and control of plague in the West Nile region (*12*). During 2008–2016, active surveillance for human plague cases occurred in 10 clinics and 2 hospitals in the Arua and Zombo Districts of the West Nile region. In addition, active community engagement with village health workers and traditional healers was undertaken to identify cases occurring in villages among persons not seeking medical care. Clinic and hospital staff received annual training on the epidemiology and clinical management of plague.

CDC-trained UVRI laboratory staff located in the town of Arua (in the Arua District) performed microbiological testing on clinical specimens, including blood cultures and bubo aspirates or sputum when available. Isolation of *Y. pestis* from clinical samples was performed on sheep blood agar, and bacterial isolates were confirmed to be *Y. pestis* by bacteriophage lysis (*13*). An acute serum sample was collected as soon as possible from patients upon their arrival at the clinic, and a convalescent serum sample was collected on days 14–28 after illness onset. Verification of *Y. pestis* cultures and all serologic testing was performed at CDC’s Division of Vector-Borne Diseases, National Center for Emerging and Zoonotic Infectious Diseases, in Fort Collins, Colorado, USA.

For purposes of this summary, we defined a confirmed plague case as clinically compatible acute illness with isolation of *Y. pestis* from a clinical specimen or with >1 positive antibody titer against the F1 antigen of *Y. pestis*, a suspected case as clinically compatible acute illness without laboratory confirmation, and a probable case as a suspected case that was epidemiologically linked to a confirmed case or a suspected case with additional nonconfirmatory laboratory evidence of plague infection. Clinical signs suggestive of plague included sudden onset of fever with painful regional lymphadenopathy (bubonic), hematemesis or hematochezia (septicemic), or cough or chest pain with hemoptysis (pneumonic). Lack of laboratory confirmation could have occurred because specimens were unavailable for testing or because specimens were negative by all available presumptive and confirmatory tests. We defined adults as persons >18 years of age. We included patients who visited facilities where active surveillance was not being performed in this study; however, we excluded patients who sought healthcare in the region but were residents of another country (i.e., nearby Democratic Republic of the Congo) because of the inability to perform follow-up. The standard treatment for suspected plague in Uganda is doxycycline or chloramphenicol; during the period covered by this surveillance summary, a concurrent treatment trial evaluating the efficacy of oral ciprofloxacin against the national standard was also being conducted (*14*).

Upon notification from health facilities or community members regarding a suspected plague case, UVRI plague program staff immediately notified local public health officials and traveled to the reporting health facility to obtain additional information on the patient. In addition, staff visited the village and patient’s home to obtain more details regarding exposure and to ascertain similar illnesses in the community. Additional persons suspected to have plague were identified and referred to the nearest health facility for assessment and care. If pneumonic plague was suspected, contact tracing and prophylaxis of exposed persons was initiated with active monitoring for at least 1 week to ensure that no additional illnesses developed. In all instances, program staff worked with village health volunteers to remind village members of the signs and symptoms of plague and the importance of prompt medical care.

Information collected for each patient was age, sex, place of residence, clinical form of plague, date of illness onset, outcome, and whether an unusual number of dead rats (a rat die-off) had been noted in the village preceding illness. We performed data management and analyses in Microsoft Excel (Microsoft, Redmond, WA, USA) and Epi Info version 7.1.1.14 (CDC, Atlanta, Georgia, USA) and used Fisher exact or χ^2^ tests for comparisons, as appropriate.

## Results

A total of 255 human plague cases were reported in the West Nile region of Uganda during 2008–2016. Overall, 140 (55%) cases were classified as suspected, 37 (15%) as probable, and 78 (31%) as confirmed, including 53 confirmed by culture only, 8 by serology only, and 17 by both culture and serology. Bacterial culture was attempted for 246 (96%) cases, and convalescent serum was available for 109 (43%) cases. Among those with negative culture results and available convalescent serum samples, 79 (89%) also had negative serologic testing results.

**Figure 1:**
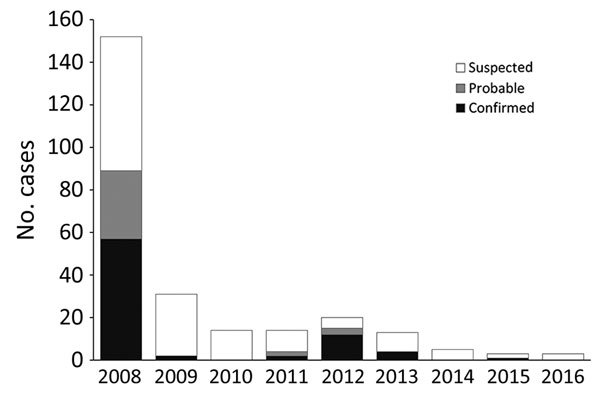
Suspected, probable, and confirmed human plague cases, by year, West Nile region, Uganda, 2008–2016.

**Figure 2:**
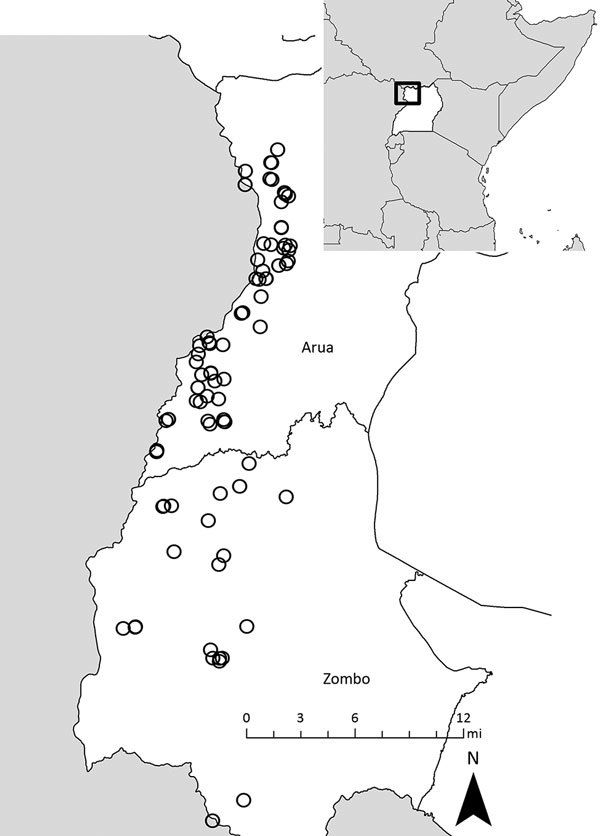
Residence locations of confirmed and probable human plague cases, by district, West Nile region, Uganda, 2008–2016. Inset shows location of Uganda in Africa.

Although yearly case counts varied widely (Figure 1), approximately three fourths of all cases, regardless of case status, occurred during October–January. Cases were identified in 130 villages in 38 parishes and 2 counties (Vurra County in Arua District and Okoro County in Zombo District; Figure 2); a total of 51 villages had ≥1 confirmed or probable plague case. Most confirmed cases (49/77; 64%) were not isolated incidents but occurred with epidemiologic linkage to >1 additional confirmed case.

**Figure 3:**
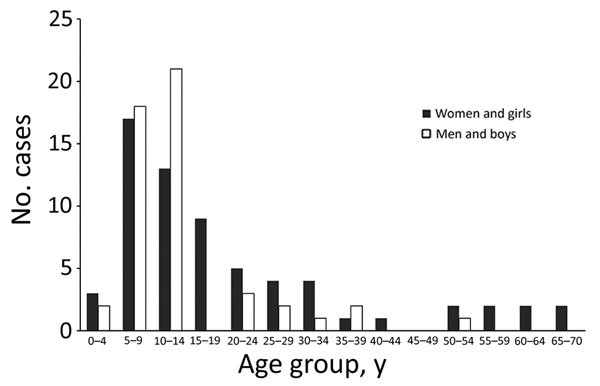
Confirmed and probable human plague cases, by sex and 5-year age group, West Nile region, Uganda, 2008–2016.

Overall median age of patients was 11 (range 1–70) years; 51% were women or girls. Among confirmed and probable cases, the sex distribution was equal among those <10 years of age, skewed toward the male sex among 10–14-year-olds, and strongly skewed toward the female sex among those >15 years of age (Figure 3). Most patients (217/255; 85%) had symptoms of bubonic plague, and the remainder were evenly split between patients with symptoms of septicemic (n = 20) or pneumonic plague (n = 18). Approximately one third of all cases, whether they were bubonic, septicemic, or pneumonic plague, were laboratory confirmed (Table).

**Table:** Case status, clinical form, and mortality rate among human plague cases, West Nile region, Uganda, 2008–2016*

Clinical form	Case status, no. patients (mortality rate, %)			
Confirmed	Probable	Suspected	Total
Bubonic	66 (26)	26 (8)	123 (7)	215 (13)
Pneumonic	6 (50)	5 (80)	7 (14)	18 (44)
Septicemic	6 (0)	6 (33)	8 (0)	20 (10)
Total	78 (26)	37 (22)	138 (7)	253 (15)

*Outcome was known for 253 of 255 total cases. The 2 suspected bubonic cases with unknown outcomes were excluded.

Among the 253 cases with outcomes reported, 37 (15%) were fatal (Table). Nine (7%) of 138 patients with suspected plague died, compared with 8 (22%) of 37 patients with probable plague and 20 (26%) of 78 patients with confirmed plague (p<0.001). As expected, the mortality rate was higher among patients with signs and symptoms of pneumonic plague; 8 (44%) of 18 patients with a pneumonic plague died compared with 27 (13%) of 215 patients with a bubonic plague and 2 (10%) of 20 patients with a septicemic plague (Table). The overall mortality rate did not differ significantly between children and adults: 22 (13%) deaths occurred among persons <18 years of age and 15 (19%) among persons >18 years of age. The case-fatality rate did not differ by sex: 18 (16%) men and boys and 19 (15%) women and girls died. Among the 19 patients who did not seek care from a clinic or hospital, 17 (89%) were eventually classified as having confirmed or probable cases, and 15 (79%) died. Among the persons who sought care from a health clinic, the time between reported illness onset and notification of public health authorities was a median of 1 day (range 0–8 days).

Information on rat die-offs was available for all but 3 cases. Rat die-offs were noted around the time of illness onset in the villages of 98 (87%) of 113 patients with confirmed or probable plague, compared with only 65 (47%) of 139 patients whose infection remained unconfirmed after laboratory testing (odds ratio 7.4, 95% CI 3.9–14.1). The positive predictive value of rat die-offs for laboratory confirmation was 60%; the negative predictive value was 83%.

## Discussion

Historical reports link the appearance of plague in Uganda to the construction of the Uganda railway during 1896–1901, a period when plague was spreading throughout many parts of the world (*9*). Plague is believed to have been present for several decades in the more southern part of the West Nile region (Okoro County, Zombo District), appearing in Vurra County of Arua District only in the late 1990s (*15*). During 2008–2016, laboratory-confirmed human plague occurred in >50 villages throughout the region. These villages were located throughout the westernmost part of the West Nile region, above the Rift Valley escarpment, where the elevation is generally >1,300 m, rainfall is high, and average temperature is relatively low compared with the neighboring lowlands (*11**,**16**–**18*). As is the case in other plague-endemic areas, human cases were highly episodic; 89 confirmed and probable cases were reported in 2008, only 4–15 in 2011–2013, and just 0–2 in the remaining years of the reporting period. Along with temporal clustering, confirmed cases tended to cluster spatially, with most occurring in association with other confirmed cases. Because all suspected cases were actively investigated, this clustering is unlikely to be explained by ascertainment bias. The demographic features, clinical forms, and seasonality of infection among these cases were similar to other reports from the region (*15**,**19*).

With a case-fatality rate of 15% overall and 26% among confirmed cases, plague mortality in the West Nile region is similar to the case-fatality rate in the United States but distinctly higher than that reported for Africa as a whole (*8**,**10**,**20*). Although the clinical features of plague can be distinctive, they are not pathognomonic. In our series, many suspected cases failed to be confirmed despite laboratory testing. Although negative results might have resulted from prior self-treatment with antimicrobial drugs in some cases, it is likely that a proportion of suspected cases were, in fact, not plague. Convalescent serum samples were available for roughly half of all culture-negative cases, and among those, nearly 90% lacked immunologic reactivity to *Y. pestis.* If many suspected cases were not plague, this fact would simultaneously explain the lower mortality rate for clinically defined cases and underscore the need for diagnostic testing when plague is suspected. Even in an outbreak setting, clinically similar illnesses might be misattributed to plague (*21*).

In the West Nile region, plague occurs most commonly among children and women, a trend that has been attributed to children and women sleeping more often on the ground and in the structures where food is stored (*15**,**20*). It is unclear, however, why boys 10–14 years of age were more affected than girls of the same age in this setting; perhaps behavioral practices common among boys approaching maturity put them in greater contact with rodents and their fleas. The male preponderance in this age group could also be simply a reflection of small number bias, given the lack of confirmed or probable cases among men and boys in the next older age group.

Molecular analyses of *Y. pestis* cultures have revealed that human illnesses in this region are caused by 2 distinct subtypes: 1 occurring predominantly in the Arua District and the other in the Zombo District (*22*). This finding, supported by multiple subtyping methods, suggests that separate enzootic cycles of *Y. pestis* occur in these respective areas, possibly with different ecologic drivers, although no substantive differences in pathogenicity or other epidemiologic features exist among illnesses in the 2 districts.

Plague is less common than many other infectious diseases, yet it retains public health importance as a disease with epidemic potential. Although potentially fatal, plague is readily treatable if appropriate therapy is initiated early. Multifaceted educational engagement efforts are needed in endemic areas to reduce *Y. pestis* disease severity and death. Although rat die-offs or rat falls have been noted throughout history to precede human outbreaks (*9*), quantitative data to support these events as predictors of human plague have been lacking. We found a strong association between recognition of a rat die-off by villagers around dates of illness onset and illnesses being confirmed as plague. Engagement of communities in plague-endemic areas to encourage the prompt notification of local authorities when a rat die-off occurs could reduce the likelihood of subsequent human infection if followed by timely application of flea control products in nearby homes. Local educational efforts through schools and the engagement of village health teams and traditional healers to recognize the signs of plague and refer villagers with suspected plague to health clinics during early illness should be sustainable interventions that could prevent larger-scale human outbreaks and reduce plague mortality rates. 

Although robust laboratory capacity was available in the region during the period we summarize, logistical challenges, such as impassable roads during the rainy season, can limit timely laboratory testing for plague in rural Africa. Sensitive and specific point-of-care diagnostic assays could improve plague diagnosis and help guide appropriate treatment in resource-limited settings.

## References

[1] Prentice MB, Rahalison L. Plague. Lancet. 2007;369:1196–207. 10.1016/S0140-6736(07)60566-2 17416264

[2] Perry RD, Fetherston JD. *Yersinia pestis*—etiologic agent of plague. Clin Microbiol Rev. 1997;10:35–66. 8993858 10.1128/cmr.10.1.35 PMC172914

[3] Gage KL, Kosoy MY. Natural history of plague: perspectives from more than a century of research. Annu Rev Entomol. 2005;50:505–28. 10.1146/annurev.ento.50.071803.130337 15471529

[4] Mead PS. *Yersinia* species (including plague). In: Bennett JE, Dolin R, Blaser MJ, editors. Principles and practice of infectious diseases. 8th ed. Philadelphia: Elsevier; 2015. p. 2607–18.

[5] Begier EM, Asiki G, Anywaine Z, Yockey B, Schriefer ME, Aleti P, et al. Pneumonic plague cluster, Uganda, 2004. Emerg Infect Dis. 2006;12:460–7. 10.3201/eid1203.051051 16704785 PMC3291454

[6] Bertherat E, Thullier P, Shako JC, England K, Koné ML, Arntzen L, et al. Lessons learned about pneumonic plague diagnosis from two outbreaks, Democratic Republic of the Congo. Emerg Infect Dis. 2011;17:778–84. 10.3201/eid1705.100029 21529384 PMC3321750

[7] Richard V, Riehm JM, Herindrainy P, Soanandrasana R, Ratsitoharina M, Rakotomanana F, et al. Pneumonic plague outbreak, Northern Madagascar, 2011. Emerg Infect Dis. 2015;21:8–15. 10.3201/eid2101.131828 25530466 PMC4285280

[8] Kugeler KJ, Staples JE, Hinckley AF, Gage KL, Mead PS. Epidemiology of human plague in the United States, 1900-2012. Emerg Infect Dis. 2015;21:16–22. 10.3201/eid2101.140564 25529546 PMC4285253

[9] Pollitzer R. Plague. Geneva: World Health Organization; 1954.

[10] Bertherat E. Plague around the world, 2010–2015. Wkly Epidemiol Rec. 2016;91:89–93.26922822

[11] Winters AM, Staples JE, Ogen-Odoi A, Mead PS, Griffith K, Owor N, et al. Spatial risk models for human plague in the West Nile region of Uganda. Am J Trop Med Hyg. 2009;80:1014–22. 19478268

[12] Centers for Disease Control and Prevention (CDC). Bubonic and pneumonic plague - Uganda, 2006. MMWR Morb Mortal Wkly Rep. 2009;58:778–81. 19629028

[13] Poland J, Dennis D. Diagnosis and clinical manifestations. Plague manual: epidemiology, distribution, surveillance, and control. Geneva: World Health Organization; 1999.

[14] Apangu T, Griffith K, Abaru J, Candini G, Apio H, Okoth F, et al. Successful treatment of human plague with oral ciprofloxacin. Emerg Infect Dis. 2017;23:553–5. 10.3201/eid2303.161212 28125398 PMC5382724

[15] Orochi Orach S. Plague outbreaks: the gender and age perspective in Okoro County, Nebbi District, Uganda. Nebbe (Uganda): Uganda Agency for Accelerated Regional Development; 2003.

[16] Eisen RJ, MacMillan K, Atiku LA, Mpanga JT, Zielinski-Gutierrez E, Graham CB, et al. Identification of risk factors for plague in the West Nile Region of Uganda. Am J Trop Med Hyg. 2014;90:1047–58. 10.4269/ajtmh.14-0035 24686743 PMC4047728

[17] MacMillan K, Enscore RE, Ogen-Odoi A, Borchert JN, Babi N, Amatre G, et al. Landscape and residential variables associated with plague-endemic villages in the West Nile region of Uganda. Am J Trop Med Hyg. 2011;84:435–42. 10.4269/ajtmh.2011.10-0571 21363983 PMC3042821

[18] Moore SM, Monaghan A, Griffith KS, Apangu T, Mead PS, Eisen RJ. Improvement of disease prediction and modeling through the use of meteorological ensembles: human plague in Uganda. PLoS One. 2012;7:e44431. 10.1371/journal.pone.0044431 23024750 PMC3443104

[19] Kilonzo BS. Plague epidemiology and control in eastern and southern Africa during the period 1978 to 1997. Cent Afr J Med. 1999;45:70–6. 10565066

[20] Davis S, Makundi RH, Machang’u RS, Leirs H. Demographic and spatio-temporal variation in human plague at a persistent focus in Tanzania. Acta Trop. 2006;100:133–41. 10.1016/j.actatropica.2006.10.006 17113555

[21] Bertherat E, Mueller MJ, Shako JC, Picardeau M. Discovery of a leptospirosis cluster amidst a pneumonic plague outbreak in a miners’ camp in the Democratic Republic of the Congo. Int J Environ Res Public Health. 2014;11:1824–33. 10.3390/ijerph110201824 24514425 PMC3945570

[22] Respicio-Kingry LB, Yockey BM, Acayo S, Kaggwa J, Apangu T, Kugeler KJ, et al. Two distinct *Yersinia pestis* populations causing plague among humans in the West Nile region of Uganda. PLoS Negl Trop Dis. 2016;10:e0004360. 10.1371/journal.pntd.0004360 26866815 PMC4750964

